# Supplementary material for: Phototriggered protein syntheses by using (7-diethylaminocoumarin-4-yl)methoxycarbonyl-caged aminoacyl tRNAs
Source: Nat Commun. 2016 Aug 17;7:12501. doi: 10.1038/ncomms12501 (PMC4992060; doi:10.1038/ncomms12501)
Supplement: Supplementary Information — Supplementary Figures 1-9, Supplementary Methods [file ncomms12501-s1.pdf]

Supplementary Information

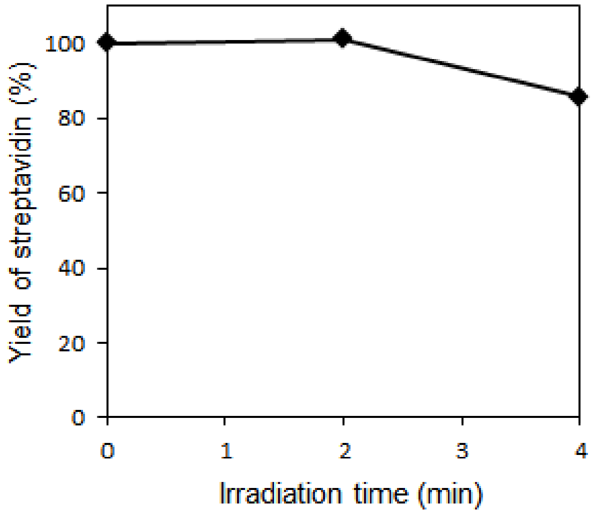

Supplementary Figure 1. Yield of streptavidin synthesized in *in vitro* translation using wild-type streptavidin mRNA after irradiation at 184 mW/cm^2 at ~405 nm.

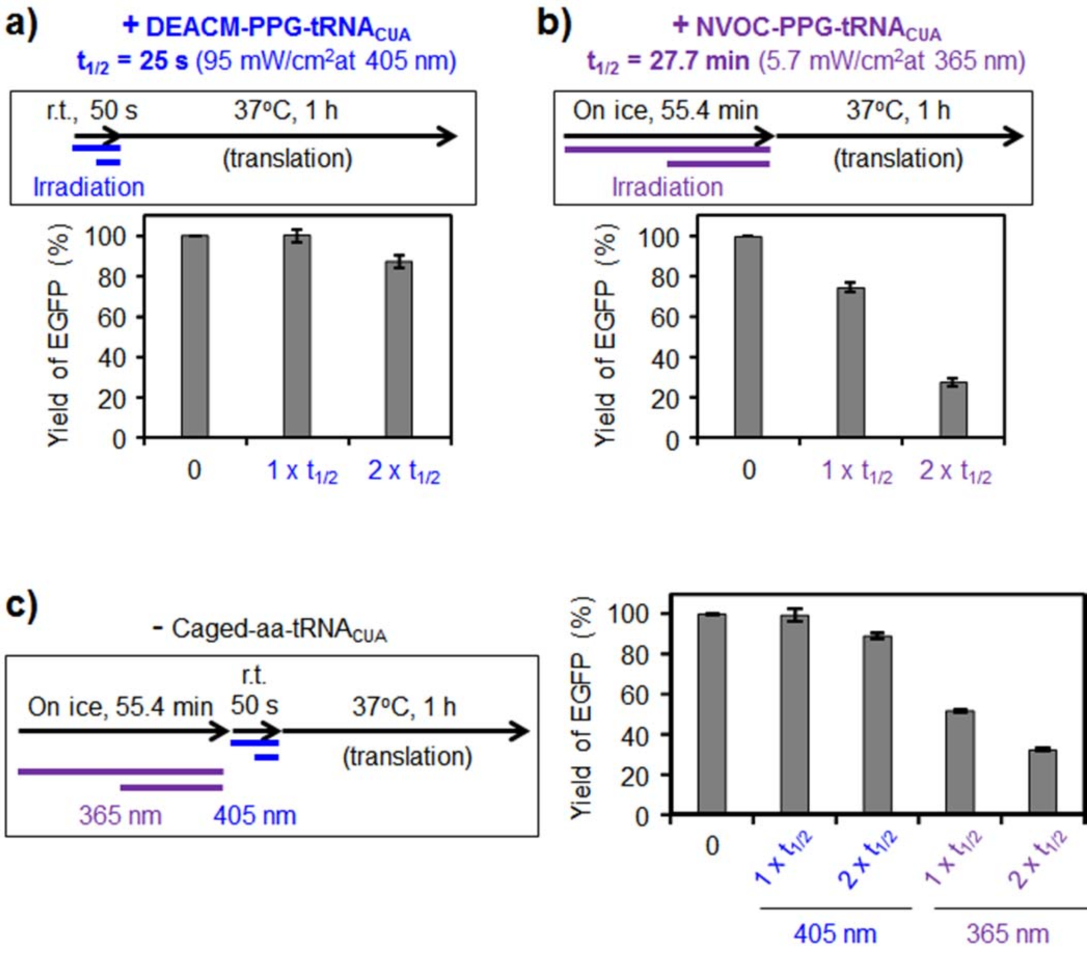

Supplementary Figure 2. Yield of EGFP synthesized in *in vitro* translation using wild-type EGFP mRNA after irradiation. A 10-µL *in vitro* translation mixture as described in the Methods was irradiated and incubated as indicated in each panel in **(a)**-**(c)**. The translation reactions were carried out in the presence of 0.1 nmol of DEACM-PPG-tRNA$_{CUA}$ **(a)** or NVOC-PPG-tRNA$_{CUA}$ **(b)**, or in the absence of caged aa-tRNA **(c)**. The caged PPG-tRNA$_{CUA}$ molecules were unnecessary for the translation of the EGFP mRNA lacking the UAG codon. These molecules were added to investigate their inhibitory effect on translation. The translation products were separated using a 15% SDS-polyacrylamide gel, and EGFP fluorescence was captured using an FLA-9000 imager (Fujifilm, Japan) with an λ_{ex}= 489 nm and λ_{em} = 508 nm. The fluorescence band intensities of EGFP were calculated using ImageJ. N = 3.

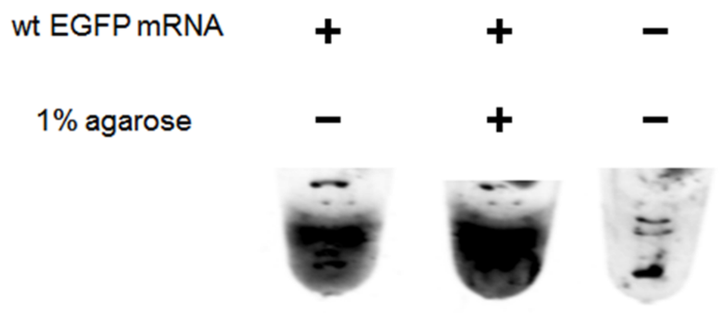

Supplementary Figure 3. Fluorescence image of EGFP synthesized in the *E.coli in vitro* translation system in the absence or presence of agarose gel. Translation mixtures including *E. coli* S30 Extract, wild-type EGFP mRNA, and 1% Seaplaque GTG agarose were incubated at 37°C for 1 h, and then the microtubes containing the translation mixtures (10 μL) were imaged using a Hitachi FMBIO III-SC01 fluorescence image analyzer (λ_{ex} = 488 nm; λ_{em} = 505–545 nm).

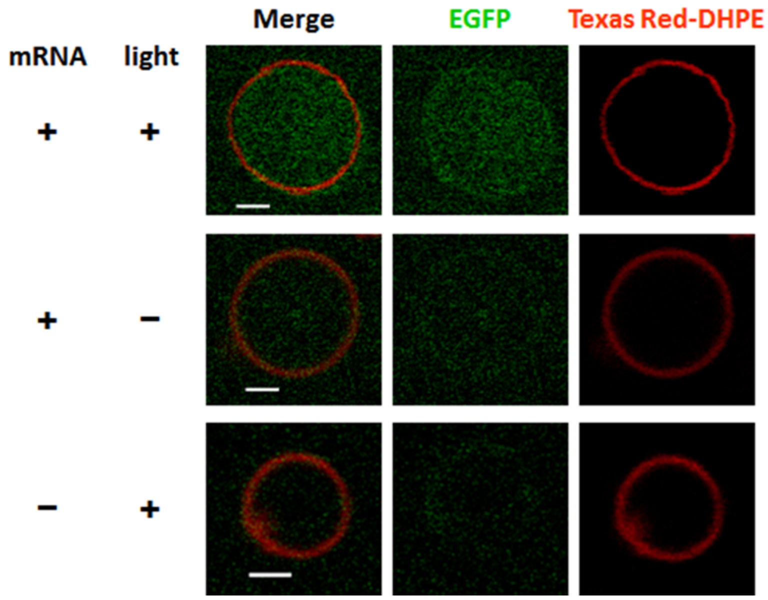

Supplementary Figure 4. Photoinduced EGFP synthesis in liposomes. Liposomes were laser-irradiated by using a confocal laser-scanning microscope (FLUOVIEW FV-1000, Olympus, Japan). Immediately after completion of the liposome preparation procedure as described in the main text, a selected liposome was irradiated with a 405-nm laser. Settings for the bleaching operation were as follows: type of bleaching: Clip Tornado; laser intensity: 20%; repetition: 35 frames. Texas Red-DHPE was used to visualize the liposome membranes. Scale bar, 2 μm.

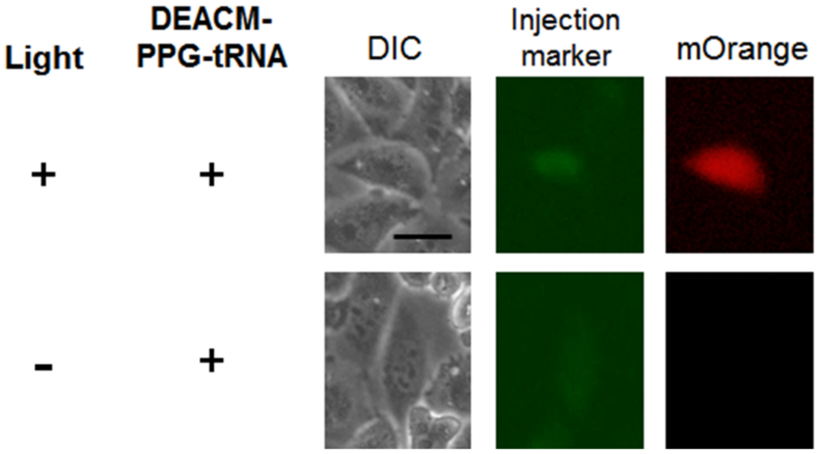

Supplementary Figure 5. Laser-induced translation by using DEACM-PPG-tRNA$_{CUA}$ and an expression vector for mOrange2 mRNA-$^{131}_{amber}$ in single CHO cells. The cells were microinjected with a solution containing DEACM-PPG-tRNA$_{CUA}$, the expression vector, and Alexa Fluor 488 C5 maleimide (upper and lower images), irradiated with laser light (upper images), and incubated for 5 h. The expression vector was prepared by mutating the Clontech pmOrange2-C1 vector at the mOrange2 codon position 131 to an amber codon. Scale bar, 25 µm.

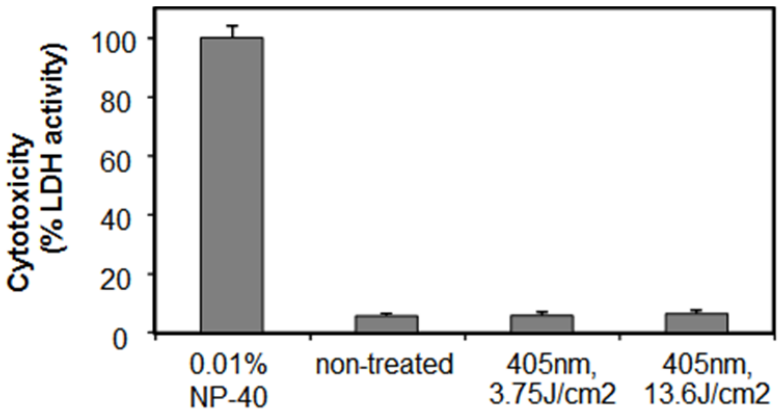

Supplementary Figure 6. Cytotoxicity after irradiation. Cytotoxicity was evaluated using CHO cells and a Cytotoxicity Detection Kit (LDH) (Roche, Switzerland). At 24 h after 3.75 J/cm^2 or 13.6 J/cm^2 irradiation at 405 nm (3.75 J/cm^2 is the same intensity as that used in the experiment in Figure 7 [30 s, 125 mW/cm^2]), an aliquot of medium was removed and mixed with the cytotoxicity detection substrate according to the manufacturer's protocol, and the absorbance at 490 nm was measured. The values were normalized by subtracting the values measured in wells containing no cells, and the values for cells that were incubated with medium containing 0.01% NP-40 were considered to represent 100% cytotoxicity.

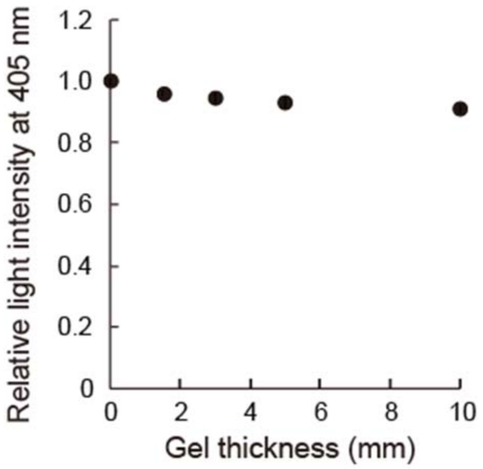

Supplementary Figure 7. Light intensity through a 0.5% agarose gel. Light intensity at 405 nm was measured using an ADCMT Optical Power Meter 8230E. The distance between the detector and the light source (a Hg-Xe lamp equipped with a long-pass filter) was constant (55 mm) in each analysis, and the gel was inserted between the detector and the light source.

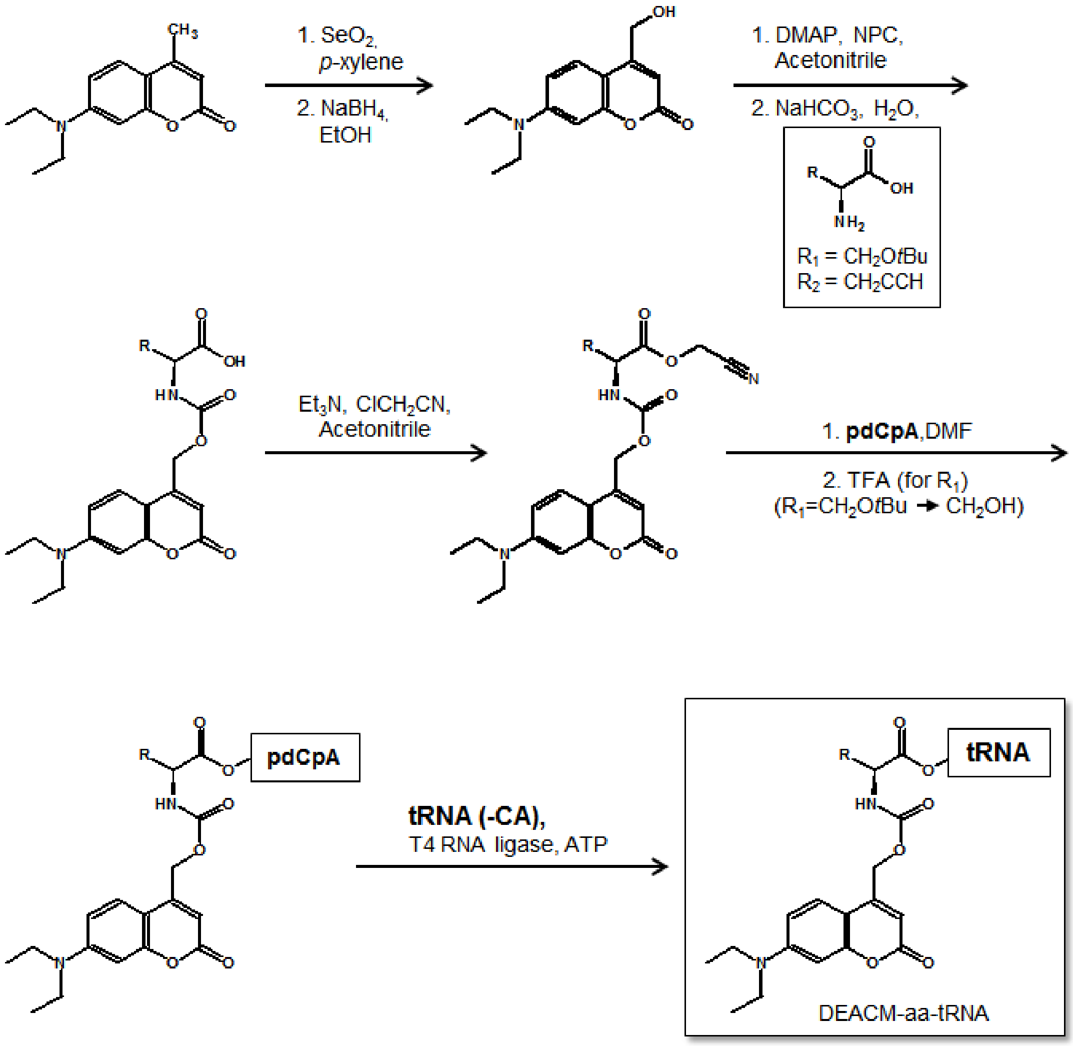

Supplementary Figure 8. Synthesis of DEACM-aa-tRNA. The amino acid (aa) moieties used were propargylglycine (PPG) and serine.

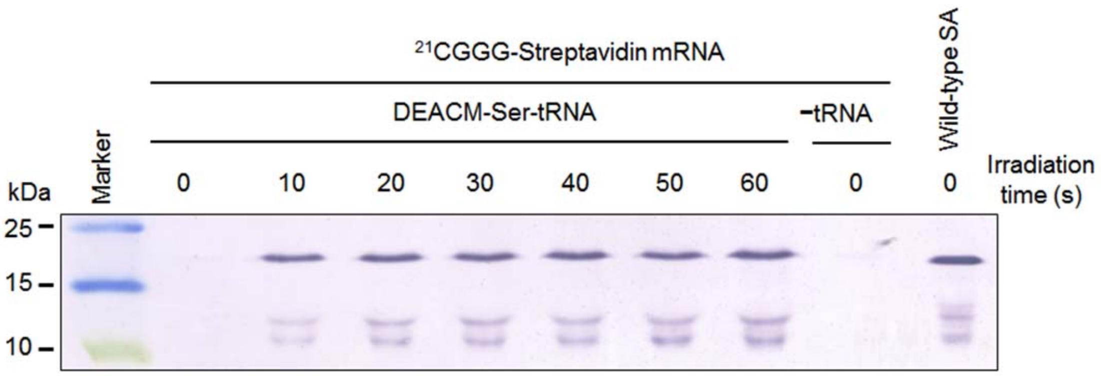

Supplementary Figure 9. Uncropped scan of the Figure 4. Frameshift suppression of the 4-base codon CGGG triggered by photoirradiation of DEACM-Ser-$tRNA_{CCCG}$ in *in vitro* translation. Synthesized proteins were detected by western blotting with an anti-T7-tag antibody. The proteins at ~11-12 kDa bands are likely to be truncated translation products (truncated streptavidin) including a T7-tag.

SUPPLEMENTARY METHODS

Synthesis of (7-diethylaminocoumarin-4-yl)methoxycarbonyl-Ser(OtBu)-OH [DEACM-Ser(OtBu)-OH]

To a stirred solution of 248 mg (1.0 mmol) of 7-diethylamino-4-hydroxymethylcoumarin (DEACM-OH) in dry acetonitrile (5 mL), we added 183 mg (1.5 mmol) of 4-dimethylaminopyridine and 241 mg (1.2 mmol) of 4-nitrophenyl chloroformate. After stirring for 4.5 h at room temperature, 5 mL of water, 252 mg (3.0 mmol) of $NaHCO_3$, and 244 mg (1.5 mmol) of *O-tert*butyl-L-serine [Ser(OtBu)] were added, and the reaction mixture was stirred at room temperature for 24 h. The mixture was concentrated under reduced pressure and the residue was purified by flash chromatography on a silica gel ($MeOH/CH_2Cl_2$ = 1/20), which yielded 169 mg (0.39 mmol, 39%) of DEACM-Ser(OtBu)-OH as a yellow solid; ^{1}H NMR (300 MHz, $CDCl_3$) δ 7.22 (d, J = 8.5, 1H), 6.50 (d, J = 8.5, 1H), 6.42 (s, 1H), 6.15 (s, 1H), 5.18 (s, 2H), 4.79 (s, 2H), 4.38 (s, 1H), 3.75 (m, 2H), 3.35 (m, 4H), 1.11 (m, 15H).

Synthesis of DEACM-Ser(OtBu) cyanomethyl ester [DEACM-Ser(OtBu)-OCM]

DEACM-Ser(OtBu)-OH [86.8 mg (0.20 mmol)] was suspended in 1 mL of acetonitrile containing 58 µL (0.80 mmol) of triethylamine. Chloroacetonitrile [38 µL (0.60 mmol)] was added to the mixture and the mixture was stirred for 12 h at room temperature and then evaporated to dryness. The residue was dissolved in ethyl acetate (10 mL) and washed sequentially with 10-mL portions of 5% aqueous $KHSO_4$ (twice), 4% aqueous $NaHCO_3$ (twice), and brine. The organic phase was dried over $MgSO_4$ and concentrated under reduced pressure, and the residue was purified through flash chromatography on a silica gel (ethyl acetate/hexane = 1/1), which yielded 28 mg (60 µmol, 30%) of DEACM-Ser(OtBu)-OCM as a yellow solid; ^{1}H NMR (300 MHz, $CDCl_3$) δ 7.28 (d, J = 8.9, 1H), 6.57 (d, J = 8.9, 1H), 6.50 (s, 1H), 6.14 (s, 1H), 5.76 (d, J = 8.8, 1H), 5.25 (m, 2H), 4.82 (m, 2H), 4.57 (m, 1H), 3.77 (m, 2H), 3.41 (m, 4H), 1.18 (m, 15H).

Synthesis of DEACM-Ser-pdCpA

DEACM-Ser(OtBu)-OCM [1.0 mg (2.1 µmol)] was added to 47 nmol of pdCpA in a tetrabutylammonium salt form in 3.5 µL of DMF. The mixture was allowed to stand for 3 h at room temperature, after which it was washed with diethyl ether (1 mL) and evaporated under reduced pressure. The tBu group was removed by dissolving the product in TFA (200 µL) for 1 h at room temperature and then the TFA was flashed off with N_2 gas. The pellet was washed with diethyl ether (1 mL) and the product was dissolved in DMSO and analyzed using HPLC and MS.

Synthesis of DEACM-PPG-OH

DEACM-OH [248 mg (1.0 mmol)] was added in 6 mL of dry acetonitrile that contained 4-nitrophenyl chloroformate [245 mg (1.2 mmol)] and 4-dimethylaminopyridine [185 mg (1.5 mmol)]. The reaction mixture was stirred at room temperature for 15 h and then 252 mg (3.0 mmol) of $NaHCO_3$ and 170 mg (1.5 mmol) of (s)-α-propargylglycine were added. The reaction mixture was stirred at room temperature for a further 2 days. $KHSO_4$ was added to reduce the pH of the mixture to 2.0–3.0, and the mixture was evaporated. The residue was extracted twice with dichloromethane and washed twice with a 10-mL portion of 5% aqueous $KHSO_4$, and twice with brine. The organic phase was dried over $MgSO_4$ and concentrated under reduced pressure, and the product was purified using silica gel chromatography

(dichloromethane/acetone/methanol = 8/8/1), which yielded 107 mg (0.28 mmol, 55.2%) of DEACM-PPG-OH as a yellow solid; ^{1}H NMR (300 MHz, $CDCl_3$) δ 7.30 (d, J = 8.0, 1H), 6.55 (d, J = 8.0, 1H), 6.50 (s, 1H), 6.28 (s, 1H), 6.08 (d, J = 11.4, 1H), 4.83 (s, 2H), 4.58 (m, 1H), 3.41 (m, 4H), 3.16 (s, 1H), 2.89 (m, 2H), 2.13 (s, 1H), 1.25 (m, 6H).

Synthesis of DEACM-PPG-OCM

DEACM-PPG-OH [50.0 mg (0.13 mmol)] was added in 1.0 mL of acetonitrile containing 36 μL (0.50 mmol) of triethylamine. Chloroacetonitrile [24 μL (0.42 mmol)] was added to the mixture and the mixture was stirred for 12 h at room temperature and then evaporated to dryness. The residue was dissolved in ethyl acetate (10 mL) and was washed sequentially with 10-mL portions of 5% aqueous $KHSO_4$ (twice), 4% aqueous $NaHCO_3$ (twice), and brine. The organic phase was dried over $MgSO_4$ and concentrated under reduced pressure. The product was purified using silica gel chromatography (dichloromethane/acetone = 20/1), which yielded 33.4 mg (80 μmol, 66.8%) of DEACM-PPG-OCM as a yellow solid; ^{1}H NMR (300 MHz, $CDCl_3$) δ 7.28 (d, J = 9.1, 1H), 6.59 (d, J = 9.1, 1H), 6.50 (s, 1H), 6.17 (s, 1H), 5.92 (m, 1H), 5.26 (s, 2H), 4.83 (d, J = 14.4, 1H), 4.81 (d, J = 14.4, 1H), 4.65 (m, 1H), 3.41(m, 4H), 2.85 (m, 2H), 2.19 (s, 1H), 1.21 (m, 6H).

Synthesis of DEACM-PPG-pdCpA

DEACM-PPG-OCM [1.0 mg (2.1 μmol)] was added to 67 nmol of pdCpA in a tetrabutylammonium salt form in 5 μL of DMF. The mixture was allowed to stand for 1 h at room temperature and then washed with 1 mL of diethyl ether (thrice) and evaporated under reduced pressure. The product was dissolved in DMSO and analyzed using HPLC and MS.
